# Workplace exposure to wood dust and the prevalence of wood-specific sensitization 

**DOI:** 10.5414/ALX01503E

**Published:** 2018-09-01

**Authors:** V. Schlünssen, T. Sigsgaard, M. Raulf-Heimsoth, S. Kespohl

**Affiliations:** 1 Department of Public Health, Unit of Environmental and Occupational Medicine, Aarhus, Denmark,; 2 Institute for Prevention and Occupational Medicine of the German Social Accident Insurance (IPA), Institute of the Ruhr-University Bochum, Germany

**Keywords:** wood dust allergy, occupational exposure, list of wood allergens, sensitization prevalence, pine wood allergy, beech wood allergy, western red cedar wood allergy, obeche wood allergy

## Abstract

Wood is processed worldwide, and occupational exposure to wood dust is affecting millions of workers. Studies have identified wood dust as a risk factor for non-malignant respiratory diseases consistent with both an allergic and a non-allergic origin. This paper summarizes our current knowledge on the importance of specific sensitization among subjects occupationally exposed to wood dust. Specific sensitization to wood dust exists, but is probably of minor importance for most wood species. In order to move the research field forward increased focus on more standardized tools for specific IgE (sIgE) diagnostics is needed and more specific tools are necessary to identify clinical relevant cases of wood dust sensitization. Moreover epidemiological studies on the occurrence of sIgE-mediated sensitization in different populations of woodworkers are needed.

**German version published in Allergologie, Vol. 35, No. 8/2012, pp. 402-412**

## Background 

Approximately 3.6 million workers in the European Union are exposed to wood dust on a regular basis ﻿[60]. Wood dust is classified as a human carcinogen [54], but beside the carcinogenic effect, a number of epidemiological studies have identified wood dust as a risk factor for asthma or asthma symptoms﻿ [24, 13, 31, 91, 93, 56], nasal impairmen﻿t [3, 51, 90], and acute or chronic impairment of lung functi﻿on [51, 74, 72, 57] all diseases consistent with both an allergic and a non-allergic origin. 

Worldwide 12,000 tree species exist, and more than 1000 of these are used for commercial purposes [54]. The major part (about 95 weigh percent) of wood is composed of cellulose, hemicellulose and lignin. The remaining 5% are numerous others high and low molecular weight organic and inorganic compounds, including proteins, which can be extracted (“wood extractives”). Examples for low molecular compounds are terpenes, terpene derivates like abietic acid, phenolic compounds, tannins, stilbenes, flavonoids, and glycosides, many with known sensitising and irritative propert﻿ies [45, 107]. Specific IgE reactivity demonstrated by immunoblots with sIgE binding to single proteins from e.g.﻿ pine [95] and locust wood dust [63] have been described. One major wood allergen, Trip s 1 in obeche wood is identified [64] until now. 

This paper summarizes our current knowledge on the importance of wood specific sensitization among subjects occupationally exposed to wood dust. 

## How common is specific sensitization against wood dust in general? 

### Case reports and clinical evaluations 

Case reports describing clinically verified asthma or rhinitis, where sIgE-mediated sensitization to wood dust is the suspected mechanism have frequently been published. Table 1 summarize wood species able to induce sIgE-mediated allergic symptoms or sensitization in exposed subjects. Most of these studies were based on single cases or clinical evaluations of asthma or rhinitis in occupationally exposed woodworkers. Altogether more than forty wood species with the ability to induce sIgE-mediated allergic symptoms have been identified. Numerous wood types are able to induce skin diseases like allergic or toxic contact dermatitis. For type IV mediated allergic contact dermatitis mostly secondary wood substances and not proteins are responsible. 

Clinical observations in patients referred to occupational depar﻿tments [59,﻿ 76, 61] indicate, that wood dust induced sIgE-mediated allergy is of importance – i.e. more than 20% of the referred patients from the wood industry are reported to be sensitized to different kinds of wood dust measured by SPT or intra-cutaneous test e.g. obeche, birch, ash, oak and beech. 

### Epidemiological studies 

A few epidemiological studies have evaluated the prevalence of sIgE-mediated sensitization to wood dust among woodworkers. 

Among 268 Swedish wood furniture workers [104] it was seen from testing of symptomatic and non-symptomatic subgroups that 13% (3 out of 23) with nasal hypersensitivity symptoms had a positive SPT to oak, beech, mahogany, birch or teak, whereas 7% (1 out of 14) from the non-symptomatic group had a positive SPT to at least one wood dust extract. Based on numbers, tested in a subgroup, the authors concluded, that 2% of wood furniture workers had wood dust allergy.**

Carosso et al. [20] found at least one positive SPT for various wood dust extracts (including walnut, obeche, douglas fir, mansonia, chestnut, poplar and oak) among nine out of 20 exposed subjects with bronchial hyperresponsiveness and asthma symptoms, whereas among 70 exposed subjects without asthma symptoms, only three had a positive SPT. None of 53 unexposed controls were sensitized. 

A Swedish study [2] among 130 woodwork teachers and 112 other teachers found no difference with regard to sIgE for wood dust (positive in two reference subjects (birch and pine) and one woodwork teacher (alder)), despite an increased frequency of self-reported wheezing among teachers exposed to wood dust. 

A recent study among 101 carpentry apprentices found 9% sensitized (SPT) to various kinds of wood extracts (14 types of wood, not specified). All apprentices with specific sensitization to wood dust were also sensitized to common aeroallergens and had rhinitis [19]. 

In a large Canadian study 1,205 Canadian sawmill workers had SPT performed for various types of wood extracts [29]. This study found 2.7% (3 out of 111) pine-exposed workers sensitized, whereas the numbers for fir/spruce sensitization were 5% (47 out of 876 fir/spruce exposed), and 7% for birch sensitization (6 out of 87) for birch exposed subjects, respectively. No unequivocal relationship between exposure to specific types of wood and prevalence of specific sensitization was seen. 

## Specific sensitization against pine wood 

Pine wood, for example *Pinus radiata* and *Pinus sylvestris*, is processed worldwide in the wood industry, and more epidemiological studies have elucidated an increased frequency of asthma symptoms, decreased lung function, and increased bronchial responsiveness among pine worker﻿s [31, 32, 92, 70] compared to low or unexposed workers. Results from the earlier mentioned Swedish [2] and Canadian [29] studies suggest sIgE against pine wood in less than 5% of the woodworkers. 

From a Danish cross-sectional study among 2,033 wood furniture workers - predominantly exposed to pine - and 474 non-exposed reference workers, a subsample comprising 365 woodworkers and 88 reference workers were clinically investigated, among others for specific sensitization to pine (*Pinus sylvestris*). Among the woodworkers 3 % had increased sIgE, 5% a positive SPT, and 6% a positive histamine release test for pine. The frequency of pine sensitization was similar among the non-exposed controls, but pine sensitization was associated with respiratory symptoms, strongest for sIgE [95, 93]. In a cross-sectional study in the same region seven ye﻿ars later [89] the prevalence of pine sensitization among woodworkers was 2% and the prevalence of wood dust sensitization was dose-dependently associated to the current level of wood dust exposure. No relation was observed between wood dust sensitization *per se* and respiratory symptoms. Pine wood sensitized workers showed a high prevalence of sensitization (73%) to cross-reactive carbohydrate determin﻿ants (CCD) [66]. Specifying sIgE-epitopes in regard of binding to proteins or glycans it was demonstrated that sera from workers reporting allergic symptoms recognized predominantly proteinogenic sIgE-epitopes on wood allergens. Woodworkers without allergic symptoms but with sIgE to wood allergens had primarily sIgE-epitopes to glycogenic﻿ structures [89, 66]. 

## Specific sensitization against beech wood 

Results from the earlier mentioned Swedish [2] study suggest a low prevalence of beech wood sensitization (no increased sIgE among 130 woodworkers). 

Another study has specifically investigated the prevalence of beech wood sensitization in the Danish wood fur﻿niture study [89, 66]. The prevalence of beech wood sensitization among current woodworkers was 3.1 %. No differences in sensitization rates were found between wood dust exposed workers and unexposed references [66], but the prevalence of wood dust sensitization was dose-dependently associated to the current level of wood dust exposure. No relation was observed between wood dust sensitization *per se* and respiratory symptoms. However, increased ORs were calculated for sIgE sensitization to beech based on proteinogenic epitopes and respiratory symptoms, although ORs were not significantly different. 

## Specific sensitization against western red cedar wood 

Western red cedar (WRC) is a well-documented cause of occupational asthma, with sensitization prevalence among exposed workers betw﻿een 4 and 13% [24, 55, 15, 28]. Occupational asthma caused by WRC has been studied extensively because it affects a vast number of woodworkers in e.g. North America and Japan. The diagnosis was confirmed in clinical investigation, often with specific bronchial provocation tests, and the aetiological agent has been identified as the low molecular weight (LMW) agent plicatic acid [21, 25]. In a single study, increased sIgE to WRC was documented among 44% (8 out of 18) with a positive specific provocation test for plicatic acid [98], but further studies have shown sIgE-mediated sensitization to be of minor importance for the aetiology﻿ of WCR asthma [101, 39, 40, 22]. So far, studies failed to clarify which specific immunologic mechanism(s) is responsible, but T-lymphocytes responding to conjugated plicatic acid seems to be present in patients with WRC asthma [38]. 

## Specific sensitization against obeche wood 

Dust from obeche wood (*Triplochiton scleroxylon*), which has a higher protein content [64] compared to e.g. pine dust is suspected to be a strong sIgE-mediated sensitizer, but has not been evaluated in epidemiological studies. Several case reports on allergic asthma have﻿ been published [18, 5, 103, 64, 37, 84, 87, 76, 61, 48]. Furthermore, Quirce at al. [84] revealed positive SPT and increased sIgE among five out of five carpenters with respiratory symptoms, and revealed similar sIgE-binding patterns of obeche extract with SDS-PAGE blot. In a small study, seven out of ten symptomatic woodworkers were sensitized and a 38 kDa class I chitinase obeche wood allergen, Trip s 1, was identified [64]. Based on allergen homology, cross-reactivity to latex allergens [102] and tamarillo fruit [103] was observed. In contrast to other wood dust sensitization, obeche wood-sensitized subjects were at risk of getting allergic symptoms also by inhalation or ingestion of non-wooden material in non-occupational settings based on Trip s 1-homolog allergens (e.g. with hevein domains). Obeche wood seemed to be a sensitizer with pronounced allergenic potency. To evaluate the airborne exposure of woodworkers to obeche wood allergens a quantification ass﻿ay was developed [65]. The assay is able to quantify allergen concentrations from 30 to 300 ng/ml. Hence exposure to airborne obeche wood allergen could be monitored in wood processing companies. Interestingly, the study demonstrated that obeche wood entities like obeche wood from Cameroon (called ayous) had less allergen content compared to obeche wood from Ghana (called wawa). Further analysis showed that the reduced allergen content in ayous wood could be ascribed to a reduced amount of major obeche wood allergen Trip s 1. This emphasises the importance of using the content of wood dust allergens, and not only the concentration of airborne wood dust, in estimated dose-response relations. 

In a clinical evaluation from Finland on occupational rhinitis [59] five out of nine patients with rhinitis verified by nasal challenge had a positive SPT for obeche wood. 

## Discussion 

It seems reasonable, that many wood species are capable of inducing sIgE-mediated sensitization via inhalation of wood dust. On the other hand it is obvious, that most of our current knowledge is based on case histories or clinical evaluations of patient series. A recently published meta-analysis [80] on wood dust exposure and asthma showed that the relative risk of asthma among exposed woodworkers was significantly higher than among the general population but the underlying pathological mechanisms are not fully elucidated, yet. There are toxic and/or lytical effects as observed for abietic acid [9] involved as well as irritative effects for terpenes [70] and immunological, sIgE-mediated effects for obeche wood allergen Trip s 1 [64]. The few epidemiological studies in the area suggest sIgE-mediated sensitization to be present, but not common, at least not for frequently used wood types in the temperate zones, for example pine, spruce and beech. Obeche wood as well as other tropical woods might be an exception and are suspected to have a strong allergenic potential, but this has to be further investigated in epidemiological studies among exposed workers. 

The epidemiological findings are not directly in line with clinical case series on wood dust-related asthma or rhinitis that reported more than 20% of the cases are sensitized to wood d﻿ust. For example, Kanerva et al. [59] found 13 out of 30 subjects with rhinitis caused by wood dust confirmed in nasal provocation tests to be positive to at least one skin prick test to wood dust. The clinical cases represent a highly selected group of workers, which may explain the difference in prevalence in the epidemiological studies and the case series. Another explanation might be the type of wood dust. 

Different wood species have different toxicological and allergenic potential due to the different chemical composition including protein content, e.g. pine wood has in general a low but variab﻿le protein content [95, 66], whereas the protein content in obeche wood is substantially higher [65]. Western red cedar has a high content of plicatic acid compared to other types of wood [27]. Tropical woods in general have a higher content of volatile and non-volatile compounds compared to wood types from ﻿other climate zones [44, 107]. 

Another limitation hampering epidemiological studies is the very heterogeneous way in which wood dust extracts are prepared and utilised. No standardised commercial available extracts are available, and most studies use in-house preparations of extracts for SPT and for detection of sIgE, making direct comparisons between studies difficult. Furthermore different methods for determination of sIgE are applied. 

A recent publication has demonstrated, that sIgE to CCDs is very frequent, at least for﻿ pine and beech dust [66]. The results of this study also suggested that sIgE epitopes to glycogenic structures were of less clinical relevance compared to proteinogenic sIgE epitopes, and the authors recommend the application of CCD tools to assess the relevance of individual wood sensitization, which has not been done in any other studies so far. 

From our knowledge no study has investigated the association between the airborne allergen concentration level and the prevalence of specific sensitization among woodworkers, and only one study has explored the association between the airborne wood dust concentration level and t﻿he prevalence of sIgE [89]. This study demonstrated a clear positive dose-dependent relation indicating that the level of exposure among woodworkers has an impact on the sensitization rate, which has also been seen in other i﻿ndustries, e.g. bakers [52]﻿ and lab animal workers [73]. 

Cross-reactivity among plant allergens has been suggested to be of importance between different types of wood (e.g. obeche and ramin [48]) between pollen and wood dust (e.g. spindle tree wood dust and mugwort pollen [47]), and finally between wood dust and other plant material, revealed for natural rubb﻿er latex and both obeche [102]and jelutong wood [108]. As described in Kespohl et al. [66] no cross reactivity was seen between beech wood and beech pollen. At the same time a high correlation between pine dust and pine pollen was revealed, possible due to common CCDs. Taken together cross reactivity indeed exist, but sensitization to pollen do not in general explain the specific sensitization reactions seen against wood dust. Furthermore specific reaction against certain types of wood dust cannot solely be explained by cross reactivity. 

In conclusion, specific sensitization to wood dust exists, but is probably of minor importance for most wood species. In order to move forward increased focus on more standardized tools for sIgE diagnostics is wanted and CCD-tools are needed to clarify the clinical relevant cases of wood dust sensitization. More epidemiological studies on the occurrence of sIgE in different populations of woodworkers are also highly needed. 

**Table 1.** Wood species described to induce sIgE-mediated allergic symptoms.

Woods	Species	Symptoms	Reference
Abiruana	*Pouteria bullata / caimito*	OA	Booth 1976 [14]
Alder	*Alnus glutinaosa*	OA	Ahman1995 [2]
Angelim Pedra	*Hymenolobium petraeum*	OA	Alday, 2005 [6]
Antiaris	*Antiaris africana*	OA	Higuero 2001 [50]
Ash	*Fracinus excelsior*	OA	Fernández-Rivas [36] Oertmann1993 [76] Spiewak 1994 [96]
Beech	*Fagus sylvatica*	OA,OR	Hernandez 1999 [46] Kespohl 2010 [66] Oertmann1993 [76] Kersten 1994 [61] Spiewak 1994 [96] Wilhelmsson 1984 [104]
Bethabara, Ipe	*Tabebuia spec.*	OA	Yacoub 2005 [109] Algranti 2005 [8]
Birch	*Betula spec.*	OA, OR	Wilhelmsson 1984 [104] Ahman 1996 [3] Ahman 1996 [4]
Cabreuva	*Myrpcarüis fastogoatris*	OA,OR, EAA	Pala 2010 [79] Baur 2000 [12] Innocenti 1991 [53]
Cedar of Lebanon	*Cedra libani*	RS	Greenberg 1972 [43]
Cedar eastern white	*Thuja occidentalis*	OA	Cartier 1986 [21] Malo 1994 [69]
Cedar western red	*Thuja plicata*	OA	Chan-Yeung 1992 [26] Lam 1983 [67] Côté 1990 [30] Pickering 1972 [81]
Cedrorana	*Cedrelinga catenaeformis Ducke*	OA, OR	Eire 2006 [35]
Cherry	*Prunus avium*	Allergy	Abendroth 1992 [1] Kersten 1994 [61] Obata 2000 [75]
Cocobolla / Palisander	*Dalbergia retusa * *Dalbergia spec.*	RS OA	Eaton 1973 [34] Godnic-Cvar 1990 [42]
Falcata wood	*Albizia falcataria*	OA	Tomioka 2006 [97]
Fernanbouc	*Caesalpinia echinata*	RS	Hausen 1990 [45]
Fir	*Abies spec.*	OA	Kespohl 2011 [62] Kersten 1994 [61]
Gaboon	*Aucoumea spec.*	OA	Kersten 1994 [61]
Imbuia	*Phoebe porosa*	OA	Jeebhay 1996 [58] Pitt 1985 [82]
Iroko, Kambala	*Chlorophora excelsa*	OA	Kersten 1994 [61] Ricciardi 2003 [88] Pickering 1972 [81] Azofra 1989 [10]
Jatoba wood	*Hymenaea courbaril*	OA	Quirce 2004 [85]
Kejaat wood	*Pterocarpus anglolensis*	OA	Ordman 1949 [77]
Limba	*Terminalia superba*	OA	Oertmann 1993 [76] Kersten 1994 [61] Wirtz 1997 [106]
Lobust wood	*Robinia pseudoacacia L.*	OA	Kespohl 2006 [63]
Mahagoni african	*Khaya anthoteca*	OA	Oertmann 1993 [76]
Mahahoni american	*Swietenia mahagoni*	OA	Kersten 1994 [61]
Macrore	*Tieghemella heckelii*	OA	Oertmann 1993 [76] Kersten 1994 [61]
Meranti	*Shorea pauciflora*	OA	Vandenplas 1996 [99] Kersten 1994 [61]
Mukali	*Aningeria robusta*	OA	Garcés 1995 [41]
Oak	*Quercus robur*	OA, OR	Abendroth 1992 [1] Aldunate 1998 [7] Malo 1995 [68] Oertmann 1993 [76] Wilhelmsson 1984 [104] Spiewak1994 [96]
Obeche	*Triplochiton scleroxylon*	OA, OR	Campo 2012 [18] Airaksinen 2008 [5] Vidal 2006 [103] Kespohl 2005 [64] Pontier 2002 [83] Ferrer 2001 [37] Quirce 2000 [84] Wirtz 1997 [106] Reijula 1994 [87] Oertmann1993 [76] Kersten1994 [61] Hinojosa1986 [48] Hinojosa1984 [49]
Olive tree	*Olea europaea*	sIgE	Campo 2010 [19]
Palisander	*Dalbergia spec.*	RS OA	Eaton 1973 [34] Godnic-Cvar 1990 [42]
Pau marfim	*Balfourodendron riedelianum*	OA	Basomba 1991 [11]
Pine	*Pinus sylvestris*	OA	Kersten 1994 [61] Dutkiewicz 2001 [33] Kespohl 2011 [62] Kespohl 2010 [66] Skovsted 2003 [95] Douwes 2001 [31] Malmström 1999 [71] Spiewak1994 [96]
Ramin	*Gonystylus bancanus*	OA	Hinojosa 1986 [48]
Sequoia	*Sequoia sempervirens*	OA	Chan-Yeung 1976 [23]
Sapelli / Sipo mahagoni	*Entandrophragma cylindricum*	OA	Kersten 1994 [61] Oertmann 1993 [76]
Soapbark	*Quillaja saponaria*	OA	Raghuprasad 1980 [86]
Spindle tree	*Eunominus europaeus*	sIgE	Herold 1991 [47]
Spruce	*Picea abies*	OA	Oertmann 1993 [76] Kersten 1994 [61] Kespohl 2011 [62]
Tali wood	*Erythophleum suaveolens*	OA	Quirce 2004 [85]
Tanganyika	*Tanganyika aningre*	OA	Paggiaro 1981 [78]
Teak	*Tectona grandis*	OA	Oertmann 1993 [76]
Walnut	*Juglans olanchana*	OA	Bush 1983 [16]
Zebrawood	*Microberlinia spec*	OA	Bush 1978 [17]

The list of relevant wood allergens was generated as update of following sources: van Kampen V et al. [100]. Sastre J and Quirce S. Sensitizing Agents Inducers of Occupational Asthma and Hypersensitivity Pneumonitis. http://eaaci.net/sections-a-igs/ig-on-occupational-allergy/allergen-list.html, Wirtz C et al. [105]. http://www.Allergom.org – wood allergens on Allergome database December 2011.OA: occupational asthma, OR: occupational rhinitis, RS: not sIgE-mediated respiratory symptoms, EAA: extrinsic allergic alveolitis, sIgE: specific IgE.
